# Socio-demographic determinants of coinfections by HIV, hepatitis B and hepatitis C viruses in central Italian prisoners

**DOI:** 10.1186/1471-2334-7-100

**Published:** 2007-08-30

**Authors:** Giuseppe La Torre, Luca Miele, Giacomina Chiaradia, Alice Mannocci, Manuela Reali, Giovanni Gasbarrini, Elisabetta De Vito, Antonio Grieco, Walter Ricciardi

**Affiliations:** 1 Institute of Hygiene – Catholic University of the Sacred Heart, Rome, Italy; 2 Institute of Internal Medicine – Catholic University of the Sacred Heart, Rome, Italy; 3 Chair of Hygiene – University of Cassino, Cassino, Italy

## Abstract

**Background:**

The coinfections HIV/HCV/HBV are an important health issue in penitentiary communities. The aim of the study was to examine HIV, HBV and HCV coinfections determinants amongst prisoners in the jails of Southern Lazio (Central Italy), in the period 1995–2000.

**Methods:**

Diagnosis of seropositivities for HIV, HBV and HCV was made using ELISA method. A multiple logistic regression analysis was conducted to verify the influence of socio-demographic factors on the HIV/HBV/HCV coinfections.

**Results:**

HIV/HCV, HBV/HCV and HIV/HBV coinfections were detected in 42 (4%), 203 (17.9%) and 31 (2.9%) inmates, respectively. These coinfections are significantly associated with the status of drug addiction (OR = 16.02; p = 0.012; OR = 4.15; p < 0.001; OR = 23.57; p = 0.002), smoking habits (OR = 3.73; p = 0.033; OR = 1.42; p = 0.088; OR = 4.25; p = 0.053) and Italian nationality (OR = 7.05; p = 0.009; OR = 2.31; p < 0.001; OR = 4.61; p = 0.04).

**Conclusion:**

The prevalence of HIV, HBV and HCV seropositivity in jails suggests that information and education programs for inmates could be useful to reduce the spread of such infections.

## Background

Human Immunodeficiency Virus (HIV) and the hepatitis C virus (HCV) or hepatitis B virus (HBV) coinfections represent a public health problem of growing importance. Because of the similar modes of spread, many people are coinfected with HIV and HCV, HIV and HBV, and in some cases with all three viruses at the same time. In particular, HCV and HIV are called the "twin epidemics": in fact, both are blood-born RNA viruses that replicate rapidly and direct blood-to-blood transmission – for example through needle sharing – is the most efficient means of transmitting both viruses [1].

Most studies show that HIV infection leads to more aggressive hepatitis C or hepatitis B and higher risk of liver damage [1]. Studies of how HCV and HBV affect HIV infection are less clear. Most research shows that HCV does not accelerate HIV disease progression, but HIV/HCV coinfection may impair immune system recovery after antiretroviral therapy has commenced [1-3].

Coinfection can complicate treatment. People with liver damage due to chronic hepatitis are more likely to experience hepatotoxicity related to anti-HIV drugs, and this represents a major concern for prison health officials dealing with infected prisoners who need treatment, for both health management and infections control.

Prisoners are considered to be at high risk of infection with HIV [6-15] and other viruses (e.g. hepatitis and other sexually transmitted infections) [16-24] due to the high proportion of risk related behaviours during the permanence within jails, in particular injecting illegal drugs, unprotected sexual relations, common use of hypodermic needles for execution of tattoos or needle sharing [25-31]. In the United States it is estimated that the 12–15% of all Americans with chronic HBV infection, 39% of those with chronic HCV and 20–26% of those with HIV infection have a history of incarceration [32].

Among the population of injection drug users, the HIV/HCV coinfection rate may be as high as 90% (HIV coinfection) and it remains high also among populations of prisoner [1-5].

A history of drug use is common in detainee populations in many countries: in the USA 73–83% of prison inmates misuse drugs [33] while in the Republic of Ireland it is estimated that 40% of prisoners use drugs. Furthermore, an Australian study determined that during their incarceration 25–44% of inmates occasionally injected illegal drugs, 14–34% engaged in occasional anal intercourse and 5–18% did both [34].

With regards to the prisoners right to health, in Italy recent modifications of disposals on execution of punishment, safety and preventive measures towards prisoners suffering from HIV/AIDS have been performed [43,44]. Modifications of penitentiary health systems have the aim of rationalising the health interventions for prisoners (preventive, curative and rehabilitative), which are guaranteed by the Constitution for health services [45].

Because of the seriousness of hepatitis B virus, hepatitis C virus, and HIV infections among inmates, it is important to know both the prevalence of these infections and the patterns of risk related behaviours in prison environments. We initiated a study concerning the spread of bloodborne viruses among the prison entrants of Southern Lazio (Italy). The aim of this study was to investigate the association between HIV and hepatitis B and C viruses coinfections and socio-demographic determinants.

## Methods

### Study design and Setting

A cross-sectional study was conducted in three jails of Southern Lazio (Cassino, Frosinone and Latina). The study was authorized by the Ministry of Justice upon the presentation of a detailed research protocol (n° 43, 23-6-2001). A pilot study of 1 month was conducted in December 2000, in order to validate the registration procedures and the assessment form [46,47]. The survey was carried out between January 2001- December 2002 and took into account all male prisoners detained in the above mentioned jails during the years 1995–2000.

The research was conducted according to the Helsinki Declaration.

### Data collection

As far as data collection, all the information (including the testing) in the study came from the patients' chart, so a retrospective collection was realised. The medical charts of the prisoners were carefully examined by a public health doctor and by two social workers. Information regarding socio-demographic characteristics, life style habits and health status of prisoners was collected. In particular, the presence of HIV, HBV and HCV infection was ascertained testing the seropositivity against these viruses using the ELISA method. This was feasible because testing against these viruses is a standard procedure in this setting, including testing inmates that both referred or did not refer to the jail's clinic for medical reason. In the present study we considered inmates for which HIV/HCV, HBV/HCV, HIV/HBV testing were simultaneously present.

Since the prisoners are a vulnerable study population, their privacy was respected by ensuring that their names and surnames were not recorded. All of the pertinent data was transferred onto a specially prepared form and afterwards transferred into a database.

### Statistical analysis

The tables describe the distribution of infections among inmates according to several characteristics. The χ^2 ^test was performed in order to investigate the association between the presence of HIV/HBV/HCV coinfections and socio-demographic determinants as well as life style habits. The Fisher exact test was used where applicable.

In order to identify risk factors associated to HIV/HCV, HBV/HCV and HIV/HBV coinfections, three multiple logistic regression analyses were performed, using the backward elimination procedure as described by Hosmer and Lemeshow [48].

The goodness of fit of the regression model was tested using the Hosmer and Lemeshow test.

The following variables were considered as covariates: nationality (not Italian as the reference group), civil status (single as the reference group), age group (age under 35 years as the reference group), educational level (low level as the reference group), number of previous detentions (first detention as the reference group), drug addiction (no addiction as the reference group), use of methadone (no use as the reference group), presence of Syphilis antibodies (VDRL) (seronegativity as the reference group), previous sexually transmitted diseases, Tine test result (negative test as the reference group), alcohol abuse (no abuse as the reference group) and smoking status (non smokers as the reference group).

The level of statistical significance was fixed at p = 0.05.

The statistical analysis was performed using the statistical software SPSS 12.0 for Windows.

## Results

### HIV/HCV coinfection

With regards to HIV/HCV coinfection, information relative to 1047 prisoners of male gender (502 from Cassino, 485 from Frosinone and 60 from Latina) were found. Table 1 shows the socio-demographic characteristics of the prisoners.

**Table 1:** Socio-demographic characteristics of prisoners, according to seropositivity against HIV/HCV, HBV/HCV and HIV/HBV

	**HIV/HCV**			**HBV/HCV**			**HIV/HBV**		
**Characteristic**	**Total (%)**	**coinfection (%)**	**p**	**Total (%)**	**coinfection (%)**	**p**	**Total (%)**	**coinfection (%)**	**p**

*Total*	1047 (100)	42 (4)		1136 (100)	203 (17.9)		1076 (100)	31 (2.9)	
									
***Jail***									
Cassino	502 (47.9)	11 (2.2)		510 (44.9)	56 (11.0)		505(46.9)	10(2.0)	
Frosinone	485 (46.3)	30 (6.2)	0.004	494 (43.5)	96 (19.4)	< 0.001	511(47.5)	19(3.7)	0.248
Latina	60 (5.8)	1 (1.7)		132 (11.6)	51 (38.6)		60 (5.6)	2 (3.3)	
Tot	1047	42		1136	203		1076	31	
									
*Nationality*									
Italian	773 (73.8)	39 (5)		834 (73.4)	32 (3.8)		787(73.1)	28(3.6)	
Foreign	274 (26.2)	3 (1.1)	0.007	302 (26.6)	171 (56.6)	< 0.001	289(26.9)	3 (1.0)	0.029
Tot	1047	42		1136	203		1076	31	
									
*Civil status*									
Single	423 (51.5)	16 (3.8)					441(51.8)	14(3.2)	
Married	240 (29.2)	5 (2.1)		468 (51.8)	105 (22.4)		247(29.0)	4(1.6)	
Cohabitant	73 (9.0)	10 (13.7)	< 0.001	275 (30.4)	36 (13.1)	0.013	74 (8.7)	7(9.5)	0.020
Separated	71 (8.6)	4 (5.6)		75 (8.3)	15 (20.0)		74(8.7)	2(2.7)	
Widower	14 (1.7)	2 (14.3)		73 (8.1)	19 (26.0)		15(1.8)	1(6.7)	
Tot	821	37		13 (1.4)	4 (30.8)		851	28	
				904	179				
									
*Age group*									
18–24	211 (20.8)	1 (0.5)		234 (23.1)	32 (12.1)		215(20.4)	0 (0)	
25–34	469 (46.4)	21 (4.5)		507 (50.0)	114 (22.5)		482(45.8)	16(3.3)	
35–44	238 (23.5)	17 (7.1)	0.003	255 (25.2)	47 (18.4)	< 0.001	245 (23.3)	13 (5.3)	0.003
≥ 45	94 (9.3)	1 (1.1)		101 (10.0)	5 (4.9)		98(9.3)	0 (0)	
Tot	1013	40		1097	198		1040	29	
									
*Educational level*									
Illiterate	33 (3.7)	0 (0)							
Elementary school	300 (38.2)	13 (4.3)		33 (3.9)	1 (3.0)		36 (4.6)	0(0)	
Jumior high school	346 (46.3)	16 (4.6)	0.535	339 (40.5)	63 (18.6)	0.018	309(39.8)	9(2.9)	0.784
Senior high school	64 (10.6)	1 (18.9)		382 (45.7)	92 (24.1)		358(46.1)	8(2.2)	
Degree	8 (1.2)	0 (0)		73 (8.7)	11 (15.1)		65(8.4)	1(1.5)	
Tot	751	39		9 (1.1)	1(11.1)		9(1.2)	0(0)	
				836	168		777	18	
									
*Num. of detentions*									
First detention	682 (68.8)	21 (3.1)		734 (68.2)	129 (17.6)		689(67.7)	16(2.3)	
> 1 detention	309 (31.2)	20 (6.5)	0.016	342 (31.8)	65 (19.0)	0.570	328(32.3)	13(4.0)	0.142
Tot	991	41		1076	194		1017	29	

In relation to age, inmates ages were distributed as follow: 20.8% of inmates were 18–24 years of age, 46.4% were 25–34 years of age, 23.5% were 35–44 years of age and 9.3% were over 44 years of age.

The majority of inmates (46.3%) had a junior high school education and only 1.2%, had an educational level higher than secondary school. 773 prisoners (73.8%) were Italian and 274 (26.2%) were of a foreign nationality. 51.5%, had never been married, 38.2% were currently married or cohabiting and 10.3% were separated or widower.

In terms of the number of detentions, for 682 prisoners (68.8%) it was the first episode, while the other 309 persons (31.2%) had at least one other previous experience of detention. HIV/HCV coinfection was diagnosed in 42 individuals (4% of tested prisoners): 11 in Cassino (2.2%), 30 in Frosinone (6.2%) and 1 in Latina (1.7%).

Statistically significant differences in HIV/HCV coinfection were found for the following variables: the jail (p = 0.004), the nationality (p = 0.007), civil status (p < 0.001), age group (p = 0.003) and the number of detentions (p = 0.016).

Table 2 shows characteristics of the 1047 prisoners. With respect to drug use, 479 prisoners (45.7%), were drug addicted at the time of incarceration and 52 (5%) were former addicted.

**Table 2:** Health status characteristics, according to seropositivity against HIV/HCV, HBV/HCV and HIV/HBV coinfection among inmates in Southern Lazio

	**HIV/HCV**				**HBV/HCV**			**HIV/HBV**	
**Characteristic**	**Total (%)**	**coinfection (%)**	**P**	**Total (%)**	**coinfection (%)**	**p**	**Total (%)**	**coinfection (%)**	**p**

*Drug addiction*									
No	516 (49.3)	2 (0.4)		574 (50.5)	44 (7.7)		531 (49.3)	2 (0.47)	
Yes	479 (45.7)	36 (7.5)		512 (45.1)	148 (28.9)		494 (45.9)	28 (5.7)	
Former	52 (5)	4 (7.7)	< 0.001	50 (4.4)	11 (22.0)	< 0.001	51 (4.7)	1 (2.0)	< 0.001
Tot	1047	42		1086	203		1076	31	
									
*Drug type*									
Heroin	429 (41)	40 (9.3)		450 (39.6)	148 (32.9)		438 (40.7)	29 (6.6)	
Cocaine	264 (25.2)	18 (6.8)		280 (24.6)	83 (29.6)		272 (25.3)	14 (5.1)	
Tot	693	58		730	231		1076	43	
									
*Use of methadone*									
No	38 (14.4)	0 (0)		261(73.1)	35 (13.4)		249 (87.1)	22 (8.8)	
Yes	245 (85.6)	32 (13.1)	0.012	96 (26.9)	95 (99.0)	0.992	38 (13.3)	0 (0)	0.057
Tot	283	32		357	130		286	22	
									
*VDRL*									
Positive	14 (1.5)	2 (14.3)		14 (1.4)	3 (21.4)		19 (1.9)	1 (5.3)	
Negative	972 (98.5)	34 (3.5)	0.09	982 (98.6)	173 (17.6)		993 (98.1)	26 (2.6)	0.479
Tot	986	36		996	176	0.71	1012	27	
									
*Previous sexually transmitted*									
*diseases*	30 (3.2)	3 (10)		32 (3.1)	8 (25)		35 (3.7)	2 (5.7)	
Yes	900 (96.8)	31 (3.4)	0.092	983 (98.8)	182 (18.5)	0.355	922 (96.3)	24 (2.6)	0.226
No	930	34		1015	190		957	26	
Tot									
									
*Tine test*									
Positive	38 (4.5)	0 (0)		41 (4.5)	8 (19.5)		39 (4.3)	2 (5.1)	
Negative	856 (95.5)	34 (4.0)		868 (95.5)	154 (17.7)		873 (95.7)	24 (2.7)	0.382
Tot	894	34	0.393	909	162	0.772	912	26	
									
*Alcohol abuse*									
Yes	95 (9.3)	6 (6.3)		103 (9.3)	21 (20.4)		99 (9.4)	5 (5.0)	
No	925 (90.7)	36 (3.9)	0.272	1006 (90.7)	178 (17.7)	0.497	950 (90.6)	25 (2.6)	0.169
Tot	1020	42		1109	199		1049	30	
									
*Smoking status*									
Yes	717 (69.6)	38 (5.3)		778 (69.5)	160 (20.6)		739(69.8)	28 (3.8)	
No	313 (30.4)	4 (1.3)	0.002	342 (30.5)	39 (11.4)	< 0.001	320(30.2)	2 (0.6)	0.004
Tot	1030	42		1120	199		1059	30	

With regards to the type of drugs used, 429 individuals (41.0%) were heroin dependent, 264 (25.2%) were cocaine addicts, while amongst the drug addicts 245 individuals (85.6%) were methadone consumers. Most individuals (54.7%) were polydrug abusers.

Table 2 shows prisoners' health characteristics with reference to their HIV and HCV coinfection status. The χ^2 ^test showed that the variables significantly associated with HIV/HCV coinfection were drug addiction (p < 0.001), use of methadone (p = 0.012) and smoking status (p = 0.002).

The results of the multiple regression analyses are presented in Table 3. In particular we found that HIV/HCV coinfection was associated with the status of drug addiction (OR = 16.02; p = 0.012), age group (OR = 2.83; p = 0.004, for people of 35–44 years), civil status (OR = 3.09; p = 0.007, for cohabitants), smoking habits (OR = 3.73; p = 0.033) and Italian nationality (OR = 7.05; p = 0.009).

**Table 3:** Factors associated with HIV/HCV, HBV/HCV and HIV/HBV coinfections among inmates in Southern Lazio

	**HIV/HCV**		**HBV/HCV**		**HIV/HBV**	
**Characteristic**	**OR (95%CI)**	**p**	**OR (95%CI)**	**p**	**OR (95%CI)**	**p**

*Drug addiction*						
No (reference group)	1		1		1	
Yes	16.02 (3.79 – 67.74)	0.012	4.15 (2.83 – 6.09)	< 0.001	23.57 (3.16 – 175.83)	0.002
						
*Age group (years)*						
< 35 (reference group)	1		1		1	
35 – 44	2.83 (1.42 – 5.61)	0.004	1.01 (0.68 – 1.49)	0.970	2.99 (1.39 – 6.53)	0.006
≥ 45	0.91 (0.11 – 7.76)	0.933	0.371 (1.14 – 0.95)	0.040	n.a.	n.a.
						
*Smoking status*						
No (reference group)	1		1		1	
Yes	3.73 (1.11 – 12.56)	0.033	1.417 (0.950 – 2.11)	0.088	4.25 (0.98 – 18.40)	0.053
						
*Nationality*						
Foreign (reference group)	1		1		1	
Italian	7.05 (1.64 – 30.31)	0.009	2.310 (1.506 – 3.54)	< 0.001	4.61 (1.07 – 19.92)	0.040
						
*Civil Status*						
Single (reference group)	1		1		1	
Cohabitant	3.09 (1.37 – 6.97)	0.007	0.68 (0.36 – 1.27)	0.227	2.84 (1.12 – 7.19)	0.028
Married	0.70 (0.23 – 2.13)	0.534	0.86 (0.56 – 1.33)	0.500	0.71 (0.20– 2.54)	0.598
Divorced	0.76 (0.23 – 2.48)	0.649	0.78 (0.25 – 2.52)	0.701	0.82 (0.21 – 2.51)	0.636
						
Goodness of fit	χ^2 ^= 0.719		χ^2 ^= 1.649		χ^2 ^= 2.234	
H-L test	p = 0.998		p = 0.895		p = 0.946	

n.a. = not applicable

### HBV/HCV coinfection

As far as HBV/HCV coinfection was concerned, information relating to 1136 prisoners of male gender was distributed as follows: 510 in Cassino, 494 in Frosinone and 132 in Latina. The HBV/HCV coinfection had been diagnosed in 203 individuals (17.9% of tested prisoners), with statistically significant differences in the percentages among the jails (p < 0.001): 56 (2.2%) in Cassino, 96 (19.43%) in Frosinone and 51 (38.64%) in Latina. With regards to nationality, 73.4% were Italian and 26.6% were foreign. Considering those persons with only coinfection, 56.6% of them were foreign and this difference was statistically significant (p < 0.001).

Concerning civil status, 51.8% had never been married, while 30.4% were currently married, 8.1% cohabitant and 9.5% were separated or widower. Statistically significant differences were found for the following variables: civil status (p = 0.013), age group (p < 0.001) and educational level (p = 0.018). In particular, most of the people (22.49%) were 25–34 years old and had junior high school degrees (24.08%) (Table 1).

In Table 2 significant differences in HBV/HCV coinfection group are shown for drug addiction (p < 0.001) and smoking status (p < 0.001).

Table 3 illustrates the factors associated with HBV-HCV coinfection, showing that coinfection is significantly associated with drug addiction (OR = 4.15, p < 0.001), age group (OR = 0.371, p = 0.04, for people over 45 years), and Italian nationality (OR = 2.31, p < 0.001).

### HIV/HBV coinfection

HIV/HBV coinfection was studied in 1076 prisoners and these numbers were distributed as follows: 505 in Cassino (46.9%), 511 in Frosinone (47.5%) and 60 (5.6%) in Latina. The prevalence rate of this coinfection was found to be 2.9%.

73.1% of the inmates were Italian and 3.56% of them had HIV/HBV coinfections.

Most of them (51.8%) had never been married, 29.0% were currently married, 8.7% were cohabitant and 10.5% were separated or widower.

HBV/HCV coinfection was detected in 5.7% of the prisoners with drug addictions, 8.89% in methadone users and 3.8% in those inmates who smoked. With regards to educational level, 667 prisoners had an elementary or junior high school degree and 17 of them had a HBV/HCV coinfection, furthermore, only 9, of the inmates had a tertiary level degree and none of them had coinfections.

Statistically significant differences were found for nationality (p = 0.029), civil status (p = 0.020), age group (p = 0.003), drug addiction (p < 0.001) and smoking status (p = 0.004). (Tables 1 and 2)

The association between the use of methadone and HIV/HCV coinfection was almost significant (p = 0.057).

The regression analysis showed that factors associated with HIV/HBV coinfection seropositivity, were drug addiction (OR = 23.57, p= 0.002), age group (OR = 2.99, p = 0.006, for people of 35–44 years, Italian nationality (OR = 4.25, p = 0.04) and civil status (OR = 2.84, p = 0.028, for cohabitants) (Table 3).

## Discussion

This cross-sectional study carried out in the jails in Southern Lazio showed a prevalence of HIV/HCV, HBV/HCV and HIV/HBV coinfections of about 4%, 18% and 3% in inmates, respectively. Data regarding these coinfections prevalence, even if remarkable, could however represent a biased estimation of the phenomenon, as screening for these infections are not mandatory for all prisoners. In our study the percentage of prisoners actually subjected to HIV testing was 47.37% of all prisoners. This particular situation could be due to the fact that all of the penitentiaries examined in our study are jails where the prisoners are confined waiting for judgement or condemned to punishments less than 5 years.

As depicted in other international studies, our study showed that HIV, HBV and HCV sero-positivities coinfections are strictly associated with the status of drug addiction, especially intravenous heroin addiction. Moreover, the spread of HIV and viral liver infections is due to needle sharing, the most important risk related factor, confirming the results of similar national [49-51] and international investigations [7,8,12,17,28,29,31,39,41,42,52-54].

Furthermore, HIV, HBV and HCV sero-positivities are influence by civil status, being a widower or cohabitant is significantly associated with these coinfections.

Finally, it is possible to consider that even if health management of HBV, HCV and HIV sero-positivities in jails appears worrisome, there is paradoxically an opportunity for these patients of receiving good treatment inside the prison environment [55].

Our study has some limitations. One weakness is information bias. The study was based on the information gathered from the clinical charts, which may not have been complete in defining inmates' health status and diagnostics. Moreover, for some variables, such as educational level, and civil status, gathered data were not complete.

It is not known whether the study population is representative of the whole populations of participating prisons. It is possible that drug addicts were more likely to be given a HIV, HBV and HCV test, in which case an overestimation of the prevalence rates of infections could occur. In this case, if we hypothesize that all prisoners not given a test were seronegative, we would find a prevalence of 2.45%, 10.9% and 11.6%, respectively.

A fundamental role in the control of infection from HIV in prison environments may be found through prevention. As prisoner populations are at high risk for all of these infections, it is necessary for public health and institutions to collaborate to develop HBV, HCV, HIV prevention programs, including immunization, health education and substance abuse treatment. It is worrisome that not all prisoners are submitted to screening of these infections, while in other countries mandatory drug and blood tests exists [56].

In waiting for a positive evolution in the preparation of a sure and immunological vaccine, the application of direct and indirect prophylaxis measures represents the more convenient way to combat the AIDS problem in prisons.

The second fundamental aspect of the preventive strategy against HIV/AIDS in the prison environment is the thorough observance of hygiene rules either general or specific (i.e., personal use of razors, teeth brush), which are often neglected, even the most elementary ones.

The overcrowding of prisons and the consequent discomfort either among the warders or among the prisoners has become one of the most urgent problems to face. As reported by the WHO guideline to the essentials in prison health, the living conditions in most prisons of the world are unhealthy and rates of infection with HIV and hepatitis are much higher than in the general population [57]. Concerns exist that prisons could serve as a reservoir for the amplification of the transmission of infectious diseases in the wider community after the release of inmates who have became infected while incarcerated [2].

Of those identified as being infected in prison, 85% of cases were associated with pre-incarceration behaviours [35,36]. The restrictive nature of the prison environment and the scarcity of clean syringes and condoms probably heighten the hazards associated with high risk activities, thus increasing the risk of transmission from infected to uninfected inmates [37].

The scientific literature shows a strong connection between infection from HIV, HBV, HCV and the group of drug-addicts in which some risk factors are surely present [38,39]. As shown in many studies, it is clear that although the jail can reduce the consumers of drugs by parenteral mean, the risk to contract the infections from HIV, HBV, and HCV would increase for people who continue to inject toxic substances [40,41].

In conclusion, our study points out that within the inmates population a resevoir of infected individuals exists, and that the jails could represent a pivotal target for primary prevention programs (for inmates, for prison guard and for the general population when an inmate leaves the jail), secondary prevention (in order to reduce the clinical risks for progression of liver disease and AIDS, and to reduce costs for management complicated infection) and tertiary prevention (to guarantee prisoners right of health).

## Conclusion

To control the diffusion of infectious diseases, and above all HIV, in prisons, implementation of operative guidelines and adequate funding are needed. It is therefore necessary to promote epidemiological studies and cost analyse of the economic impact relating to the public health of prisoners with coinfection management, in order to know the real burden of the problem.

## Competing interests

The author(s) declare that they have no competing interests.

## Authors' contributions

All of the authors participated in the establishment of the research. GLT, EDV and WR conceived the study, participated in its design and coordination. GLT, GC and AM performed the statistical analysis. GLT, LM, MR and GC wrote the initial draft of the manuscript, which GLT, GG and AG edited. All authors read and approved the final manuscript.

## Pre-publication history

The pre-publication history for this paper can be accessed here:


